# Supplementary material for: Environmental and genetic predictors of human cardiovascular ageing
Source: Nat Commun. 2023 Aug 21;14:4941. doi: 10.1038/s41467-023-40566-6 (PMC10442405; doi:10.1038/s41467-023-40566-6)
Supplement: Supplementary file 1 — Supplementary Information [file 41467_2023_40566_MOESM1_ESM.pdf]

Supplementary Materials

Online methods

The code for each analysis step including image processing, imputation, CatBoost training, risk factor and outcome modelling, and all genetic analyses are presented in Notebook format on GitHub (https://github.com/ImperialCollegeLondon/cardiovascular_ageing).

Imputation of data

We used imputation for handling missing values in image-derived phenotypes using multiple imputations with predictive mean matching, with 5 imputations and 5 iterations from the R package `MICE`.[1] For continuous risk factor analysis (i.e. associations of age-delta with alcohol and smoking consumption, serum levels of low-density lipoprotein, triglycerides and apolipoprotein B and telomere length), we included participants with complete data only. Statistical tests used were two-sided unless otherwise specified.

Baseline participant characteristics and study flowchart

Baseline characteristics of the population are shown in Supplementary Figure 1 and a flow chart of analysis steps are shown in Supplementary Figure 2.

	Female (n = 20502)<br>Mean ± SD, or n (%)	Male (n = 18947)<br>Mean ± SD, or n (%)
**Baseline characteristics**		
Age at time of MRI (years)	63.0 (± 7.41)	64.3 (± 7.67)
Race, Caucasian, n (%)	17427 (85.0%)	16275 (85.9%)
Body Mass Index (kg/m^2)	26.1 (± 4.56)	27.1 (± 3.77)
Systolic Blood Pressure (mmHg)	135 (± 19)	141 (± 17)
Diastolic Blood Pressure (mmHg)	77 (± 10)	81 (± 10)
Pulse rate (bpm)	73 (± 12)	69 (± 12)
Alcohol intake, n (%)	11725 (57%)	13036 (69%)
Smoking amount, n (%)	4237 (21%)	5051 (27%)
Diabetes Mellitus, n (%)	910 (4%)	1566 (8%)
Hypertension, n (%)	4714 (23%)	6338 (33%)
Obesity, n (%)	3476 (17%)	3615 (19%)
Coronary Artery Disease, n (%)	694 (3%)	1965 (10%)
Hypercholesterolaemia, n (%)	2777 (14%)	4709 (25%)
**Cardiac characteristics from CMR**		
LV end-diastolic volume (ml)	129 (± 22.8)	168 (± 32.4)
LV end-systolic volume (ml)	50.3 (± 12.6)	71.2 (± 19.7)
LV stroke volume (ml)	78.8 (± 14.6)	96.6 (± 19.4)
LV ejection fraction (%)	61.2 (± 5.62)	57.8 (± 6.25)
LV cardiac output (ml)	4.99 (± 1.09)	5.89 (± 1.27)
LV mass (ml)	70.8 (± 12.4)	102 (± 18.6)
RV end-diastolic volume (ml)	134 (± 24.4)	180 (± 33.5)
RV end-systolic volume (ml)	54.9 (± 13.5)	81.1 (± 19.6)
RV stroke volume (ml)	79.3 (± 15.2)	99.3 (± 20.2)
RV ejection fraction (%)	59.2 (± 5.77)	55.2 (± 6.03)
LA maximum volume (ml)	67.7 (± 19.4)	78.5 (± 26.1)
LA minimum volume (ml)	26.7 (± 12.3)	32.5 (± 18.4)
LA stroke volume (ml)	41.0 (± 10.2)	46.0 (± 12.9)
LA ejection fraction (%)	61.8 (± 8.87)	60.4 (± 10.2)
RA maximum volume (ml)	75.8 (± 20.1)	97.9 (± 30.3)
RA minimum volume (ml)	38.5 (± 13.0)	54.7 (± 21.4)
RA stroke volume (ml)	37.3 (± 11.4)	43.2 (± 14.6)
RA ejection fraction (%)	49.5 (± 8.98)	44.7 (± 9.31)
Ascending aorta maximum area (mm^2)	786 (± 162)	926 (± 192)
Ascending aorta minimum area (mm^2)	714 (± 160)	843 (± 187)
Ascending aorta distensibility (10^{-3}.mmHg^{-1})	1.78 (± 1.39)	1.85 (± 1.22)
Descending aorta maximum area (mm^2)	427 (± 74.2)	527 (± 92.7)
Descending aorta minimum area (mm^2)	374 (± 71.1)	467 (± 88.2)
Descending aorta distensibility (10^{-3}.mmHg^{-1})	2.43 (± 1.63)	2.39 (± 1.37)
Peak longitudinal strain rate (s^{-1})	1.74 (± 0.633)	1.53 (± 0.566)
Peak radial strain rate (s^{-1})	-6.02 (± 2.09)	-5.24 (± 2.00)
Interventricular Septal T1 (ms)	942 (± 47.6)	923 (± 43.8)

Supplementary Figure 1. Baseline participant characteristics. A summary of clinical and imaging characteristics in the UK Biobank population stratified by sex. Abbreviations: LA, left atrium; LV, left ventricle; RA, right atrium; RV, right ventricle; T1, longitudinal relaxation time of tissue.

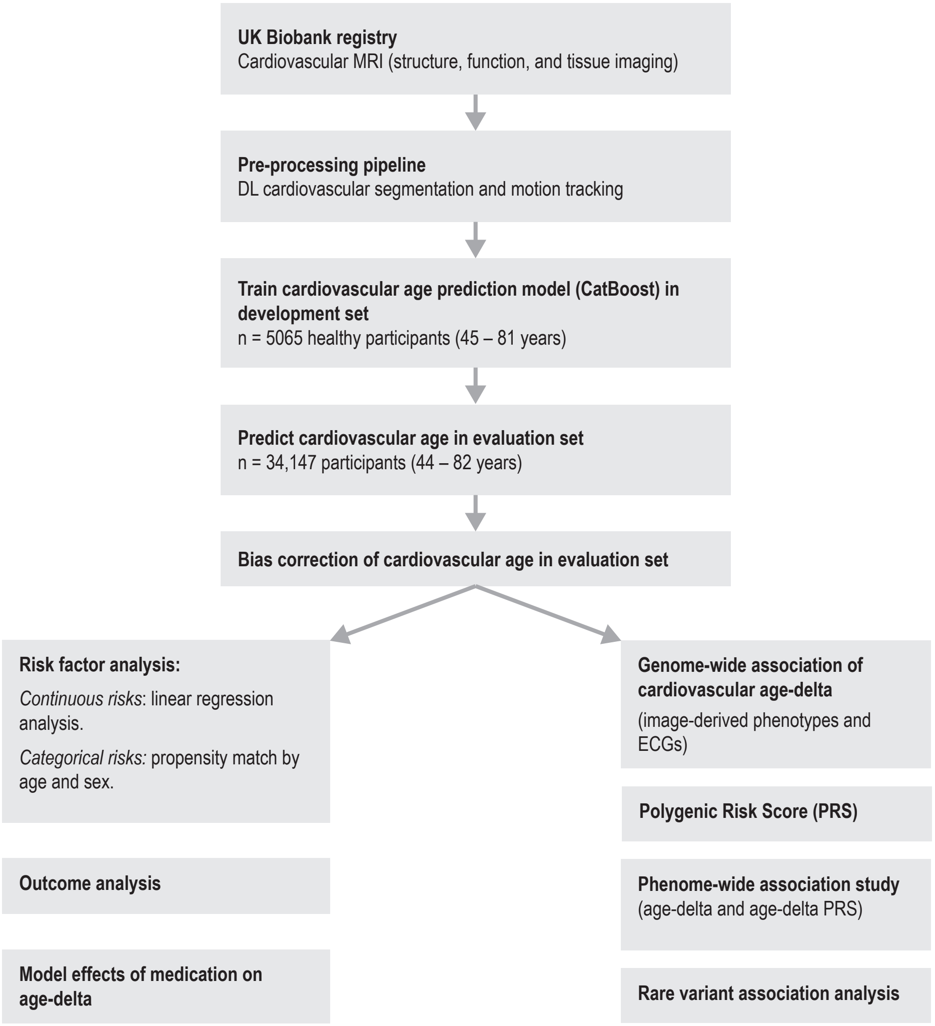

Supplementary Figure 2. Study flowchart. A summary of the main steps in our analysis of cardiovascular imaging traits, prediction of age-delta and genetic/environmental associations. MRI, Magnetic Resonance Imaging; DL, Deep Learning; ECG, electrocardiogram

Performance of predictive models

Comparison of algorithms: In Supplementary Table 1, we present an age prediction performance comparison among distinct algorithms evaluated on a holdout set of healthy participants. The CatBoost gradient boosting framework was considered as it natively handles categorical variables, uses ordered boosting to address overfitting, and is rapid to execute at scale on a graphics processing unit (GPU). Here we compare CatBoost, used in the analysis of this paper, with ridge regression, least absolute shrinkage and selection operator (LASSO), elastic net and random forests algorithms. We also report the mean absolute error (MAE), mean squared error (MSE) and coefficient of determination (R^2), with their standard errors presented in parenthesis (for the MAE and MSE metrics). The CatBoost model had the best performance metrics. Supplementary Figure 3 demonstrates the prediction using this model on the healthy participant holdout set, and Supplementary Figure 4 demonstrates associations of imaging phenotypes with age in the full development set, and represents the changes in structure and function found in healthy cardiovascular ageing.

Supplementary Table 1. Age prediction performance comparison across algorithms with standard errors presented in parenthesis.

	MAE	MSE	R^2
CatBoost	4.21 (0.10)	27.78 (1.18)	0.49
Ridge	4.32 (0.10)	29.80 (1.32)	0.46
LASSO	4.33 (0.10)	29.43 (1.30)	0.46
Elastic net	4.33 (0.10)	29.50 (1.30)	0.46
Random forests	4.61 (0.10)	32.25 (1.29)	0.41

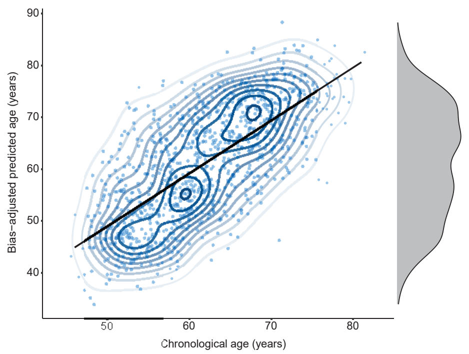

Supplementary Figure 3. The relationship between predicted and chronological cardiovascular age in the healthy training test set. Ages jittered, density contours, linear model with 95% confidence intervals, and a marginal density plot, (n=1044).

Performance of system-based models and multimodality models: In order to assess the physiological inter-relatedness between the heart and the vasculature, we trained two CatBoost models using either cardiac imaging features or vascular imaging features to compare the predictive performance with each other and with our standard cardiovascular imaging-based model. We demonstrate that a model using cardiac features only (and sex) yielded an R^2 of 0.34, whilst a model using vascular features only (and sex) obtained an R^2 of 0.36, both being a substantial drop from the R^2 of 0.49 obtained when jointly analysing both sets of features to predict cardiovascular age (Supplementary Table 2). In order to assess the performance of a multimodality model that also incorporates simple electrocardiograpic (ECG) data, we added QRS interval duration, QTc interval duration and ventricular rate as features to our existing imaging-based model (Supplementary Table 2). This showed that low dimensional pre-defined ECG features did not improve the prediction performance of the model.

Covariate shift

Measurement and prediction error in the target population may dilute the performance of the model in estimating "true" cardiovascular age and there is no measurable ground truth for biological ageing available. The validation of the model is made through correlations between age-delta and relevant clinical characteristics, health outcomes or behaviour. Here we used t-Distributed Stochastic Neighbor Embedding (t-SNE) on the covariate distributions in the healthy development set and evaluation set to assess for any domain shifts that might lead to unreliable predictions. The t-SNE visualisation (Supplementary Figure 5) represents the features used to train/evaluate the CatBoost model on 1044 participants of the test subset of the healthy development set and the 34,145 participants of the evaluation set. For visualisation purposes, the evaluation set has been

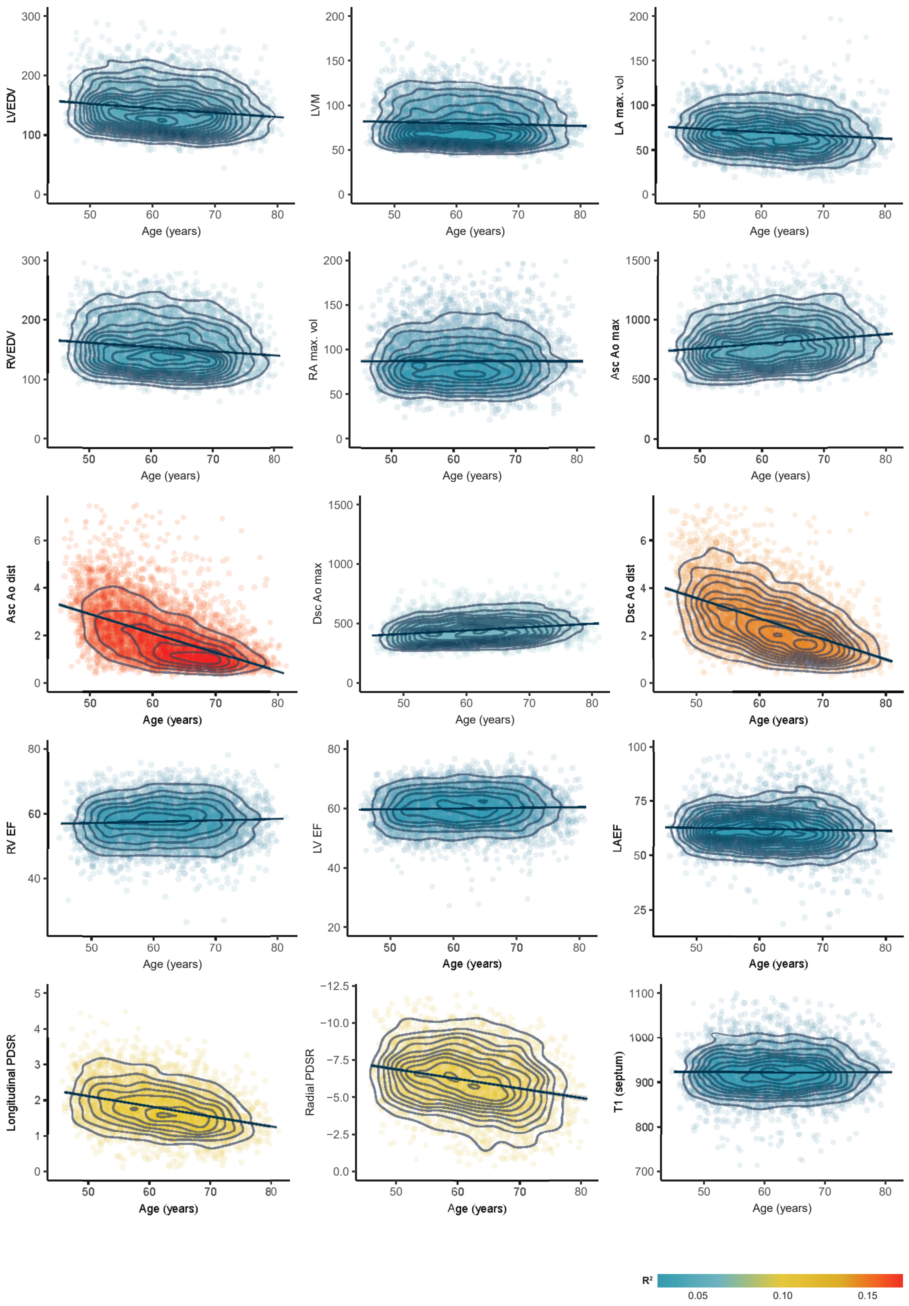

Supplementary Figure 4. Image phenotype associations with chronological age in healthy individuals. In total we measured 126 image-derived phenotypes including temporal motion analysis of cine imaging. A selection of 15 representative phenotypes of volumes, function, and tissue characterisation are shown with their relationship to chronological age at the time of imaging. (n=5296 subjects determined to be "healthy", ages jittered, density contours, point colours represent coefficient of determination (R^2)). LVEDV, left ventricular end diastolic volume; LVM, left ventricular mass; LA max. vol, left atrial maximum volume; RVEDV, right ventricular end diastolic volume; RA max. vol, right atrial maximum volume; Asc/Dsc Ao max., ascending/descending aortic maximal cross-sectional area; Asc/Dsc Ao dist., ascending/descending aortic distensibility; RV/LVEF, right/left ventricular ejection fraction; LAEF, left atrial ejection fraction; PDSR, peak diastolic strain rate; T1, longitudinal relaxation time of tissue.

randomly sampled without replacement to match the size of the healthy development set in the plots (n = 1044 participants). Additionally, we color each participant according to their bias-corrected predicted age. The model predictions and space occupied by the features are comparable between the sets, suggesting no major domain shift between the two.

Supplementary Table 2. Age prediction performance comparison across CatBoost algorithms using varying features, standard errors presented in parenthesis.

	MAE	MSE	R^2
Cardiac-only imaging features	4.89 (0.11)	36.37 (1.49)	0.34
Vascular-only imaging features	4.78 (0.11)	34.81 (1.44)	0.36
All imaging features	4.21 (0.10)	27.78 (1.18)	0.49
All imaging and simple ECG features	4.29 (0.11)	28.55 (1.30)	0.48

Additionally, in Supplementary Figure 6, we present a kernel density estimation (KDE) plot, with its bandwidth hyperparameter chosen by data splitting, as well as a density histogram of the bias-corrected age deltas calculated on 1044 participants of the test subset of the healthy development set and the 34,145 participants of the evaluation set. On the KDE plot, we additionally plot vertical lines representing the means of healthy development set and the evaluation set. The plots demonstrate that the evaluation set age-deltas have a similar distribution to the healthy development set age-deltas and have an expected shift towards a higher predicted age.

Finally, note that performance metrics used for evaluating age prediction models depend on cohort and study-specific data characteristics, and cannot be directly compared across different studies.[2]

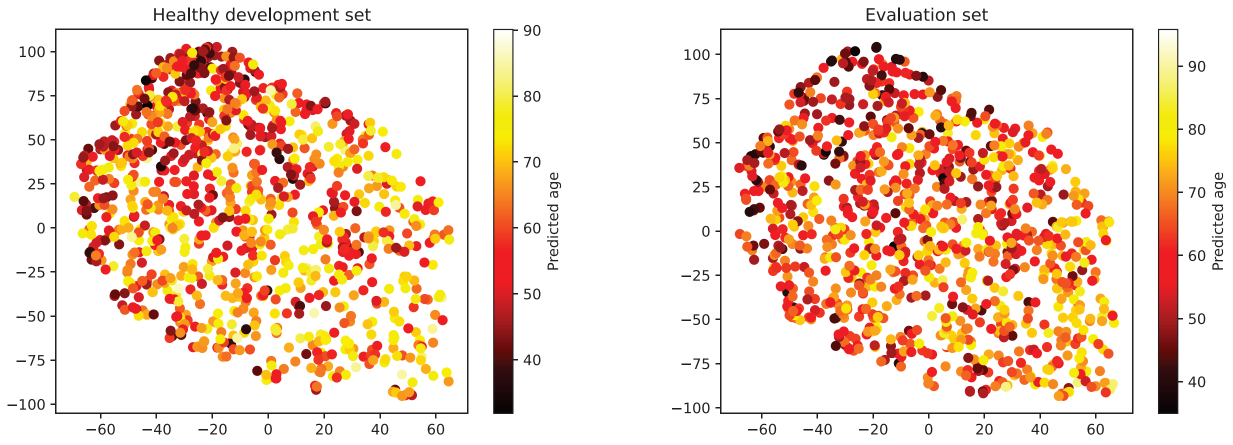

Supplementary Figure 5. T-distributed stochastic neighbor embedding (t-SNE) representation of the features used to train/evaluate the CatBoost model on participants of the test subset of the healthy development set (n = 1044) and participants of the evaluation set (n = 34,145); for visualisation purposes, the evaluation set has been randomly sampled to have the same number of plotted participants as the healthy development set. Each participant is assigned a colour according to their bias-corrected predicted age.

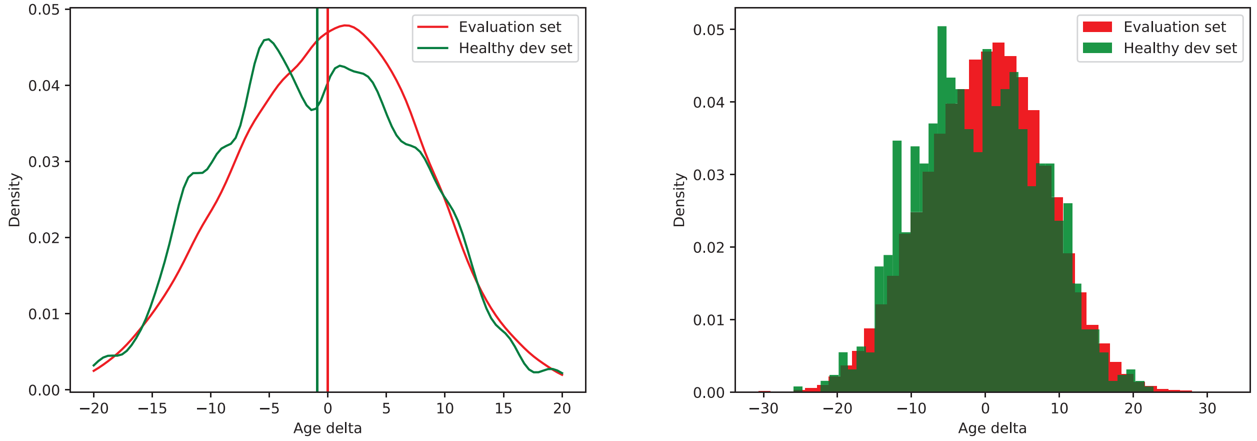

Supplementary Figure 6. Kernel density estimation (KDE) and density histogram of the bias corrected age deltas calculated on participants of the test subset of the healthy development set (n = 1044) and participants of the evaluation set (n = 34,145). On the KDE plot, two vertical lines represent the means of healthy development set and the evaluation set.

SNP effect sizes

We assessed the absolute effect size per standard deviation for each genome-wide significant SNP across three independent GWASs (Supplementary Figure 7). There were no SNPs in common between the three GWASs.

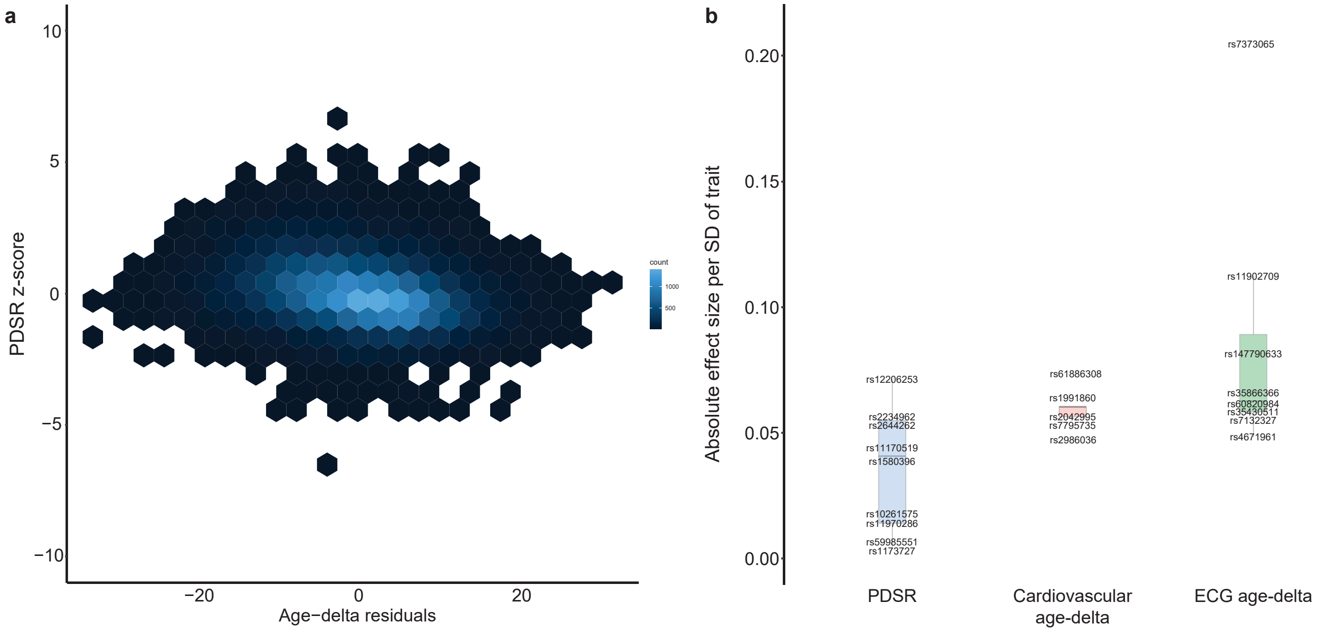

Supplementary Figure 7. Standardised effect sizes for significant SNPs. a. Hex plot of age-delta residuals and underlying trait z-scores. **b.** The absolute effect size per standard deviation for each genome-wide significant SNP across three independent GWASs. Each GWAS discovered different SNPs and are labelled by reference SNP cluster ID (RSID). Boxes show median and inter-quartile range (IQR), and whiskers show 1.5 x IQR. PDSR, peak diastolic strain rate; ECG, electrocardiogram; SD, standard deviation.

Risk factor and outcome modelling

Definition of healthy populations, evaluation set population and disease groups in the UK Biobank: Age is defined as age of participant at the date of the cardiac magnetic resonance (CMR) study, and computed as the number of years between year of birth (UK Biobank data-field 34, https://biobank.ctsu.ox.ac.uk/crystal/field.cgi?id=34) and date of CMR (UK Biobank

data-field 53, instance 2, https://biobank.ctsu.ox.ac.uk/showcase/field.cgi?id=53). Sex of participant is determined using UK Biobank data-field 31, (https://biobank.ctsu.ox.ac.uk/crystal/field.cgi?id=31).

Disease definitions were treated as binary traits, and within the UK Biobank were defined based on records from hospital episode statistic (HES) data and self-reported data. If any of the ICD9/ICD10 codes and operation or procedure codes where relevant (for HES data), and any of the self-reported codes (UK Biobank data-field 20002) are reported at any time, we considered the participant to have the disease in question.

In order to define a healthy population relevant to our study of cardiovascular ageing, the UK Biobank participants with CMR were filtered to exclude those with cardiac disease, chronic respiratory disease and metabolic disease (hypercholesterolaemia, diabetes and obesity) using codes defined in Supplementary Table 3, and BMI <30. The remaining participants with CMR were held out to comprise an evaluation set.

Groups of various relevant risk factors were also defined using the codes listed in Supplementary Table 3. For the clinical groups (diabetes, obesity, hypertension and coronary artery disease), participants could be part of more than one group. Restricting participants to mutually exclusive disease groups resulted in very small sample sizes without common co-morbidities. Telomere length, serum levels of low-density lipoprotein, triglycerides, apolipoprotein B, smoking and alcohol consumption were obtained using codes documented in Supplementary Table 3. Additionally, alcohol was computed as grams consumed per day[3].

Definition of health outcomes: In order to obtain date of clinical health outcome, the "first-occurrence" field (UK Biobank category-field 1712) was used. Time to event was then calculated as number of days between CMR and event. If no event had occurred, participants were censored and time to last data refresh (2021-02-18) used. The primary clinical outcome used was a composite of major adverse cardiovascular events (MACE) and death attributed to cardiovascular cause. Codes used for these definitions are detailed in Supplementary Table 4. Clinical outcomes for 34,133 participants were analysed. Participants had a median time to event or censor of 3.0 years (interquartile range: 2.1-4.4 years) with a total of 732 composite clinical events reported.

Supplementary Table 3. A list of diagnostic codes used to define risk factors in the UK Biobank analysis.

Title	ICD10 Code	ICD9 Code	Self-Reported Code	Operation Code	UK Biobank Field
**Hypercholesterolaemia**	E78	27202, 27209, 27200, 2720	1473		
**Diabetes Mellitus**	E10-E14		2443, 1220, 1221, 1222, 1223, 1276, 1468, 1607		
**Obesity**					21001
**General presence of cardiac disease**	I00-I25, I30-I52				
**Hypertension**	I100, I110, I119, I120, I129, I130, I131, I132, I139, I150, I51, I152, I159	4010, 4011, 4019, 4039	1065, 1072, 1073		
**Coronary Artery Disease**	I21-I25	4109, 4119	1074, 1075	1523, 1070, 1071, 1095	
**Chronic respiratory disease**	J40, J410, J411, J418, J42, J431, J432, J438, J439, J440, J441, J448, J449, J450, J451, J458, J459, J46, J47	4241	1490, 1586		
**z-adjusted Telomere Length**					22192
**Smoking**					20161
**Alcohol**					4407, 4418, 4429, 4440, 4451, 4462, 1568, 1578, 1588, 1598, 1608, 5364
**Low density lipoprotein**					30780
**Triglycerides**					30870
**Apolipoprotein B**					30640

Supplementary Table 4. A list of diagnostic codes used to define major adverse cardiovascular event (MACE) outcomes in the UK Biobank analysis.

Title	ICD10 Code	ICD9 Code	Self-Reported Code	Operation Code	UK Biobank Field
**Stroke**	I60-I69		6150		
**Arrhythmia**	I440, I441, I442, I443, I444, I445, I446, I447, I450, I451, I452, I453, I454, I455, I456, I458, I459, I460, I461, I469, I470, I471, I472, I479, I480, I481, I482, I483, I484, I489, I490, I491, I492, I493, I494, I495, I498, I499	4260, 4261, 4263, 4264, 4265, 4266, 4267, 4269, 4270, 4271, 4272, 4273, 4274, 4275, 4276, 4278, 4279			
**Heart Failure**	I255, I420, I421, I422, I423, I424, I425, I426, I427, I428, I429, I430, I431, I432, I438, I500, I501,I509	4251, 4254, 4280,4281,4289	1076, 1079		
**Cardiovascular Death**	Starts with "I"				40000

PheWAS and risk factor analysis methods

PheWAS: Phenome wide analysis of age-delta and age-delta polygenic risk score have been performed on a range of phenotypes and outcomes. Logistic regression was performed on categorical and binary variables and linear regression on continuous variables, using each of these traits as the dependent variable and age-delta or age-delta PRS as a predictor. These associations were made while adjusting for sex, age, age^2 and the first ten genetic principal components, giving the following model:

$$\text{Trait} \sim \text{Age delta or Age delta PRS} + \text{Age} + \text{Age}^2 + \text{Sex} + \text{Principal components 1-10} \quad (1)$$

For the age-delta PheWAS, we tested associations between a range of diseases classified by PheCodes (1149 traits) and found 5 reaching the significant Bonferroni threshold for multiple testing ($P < 4.4 \times 10^{-5}$). We have enriched the age-delta PRS PheWAS with a broader unbiased range of physiological measures, biomarkers and outcomes from the UKB (2407 traits including previously stated PheCodes) and found 12 significant associations when using the Bonferroni correction for multiple testing ($P < 2.1 \times 10^{-5}$), which corresponds to 8 unique traits.

Investigating associations with established risk factors: For categorical risk factor groups (i.e. presence or absence of disease), participants with the risk factor ("disease participants") were 1:1 propensity matched with participants without the risk factor ("control participants"), by age and sex. This was performed in R using the package `matchit`.[4] The imbalance of age and sex between both samples was first assessed and quantified using a student's t-test. Next, each disease participant was paired with a control participant with the closest logistic regression propensity score through nearest neighbor matching. The distribution of age and sex was again compared between groups, and additionally propensity scores plotted to visually assess matches qualitatively. Following adjustment for age, age^2 and sex, a linear regression model was used to test the association between cardiovascular age-delta and presence of disease as follows:

$$\text{Age delta} \sim \text{Disease (0/1)} + \text{Age} + \text{Age}^2 + \text{Sex} \quad (2)$$

where the coefficient of "Disease" is the effect of having the disease in terms of age-delta years.

For continuous risk variables (i.e. alcohol and smoking consumption, serum levels of low-density lipoprotein, triglycerides and apolipoprotein B, and telomere length), we fit a linear regression model in all participants of the test data set that had complete data for these variables to assess the association between cardiovascular age-delta and risk factor as follows:

$$\text{Age delta} \sim \text{Continuous risk factor (e.g. smoking pack years)} + \text{Age} + \text{Age}^2 + \text{Sex} \quad (3)$$

where the coefficient of "continuous risk factor" is the effect of an increase in unit of continuous risk factor in terms of age-delta years. We report both standardised beta coefficients in Figure 6c, to enable comparison between risk factors, and non-standardised coefficients in the text of the results section. We performed correction for multiple testing using a false discovery rate controlling procedure[5], aiming to control the false discovery rate at $\alpha = 0.05$.

Association of cardiovascular age-delta with outcomes: We assessed the association of cardiovascular age-delta with prospective cardiovascular events. Non-standard covariates are defined as described in Supplementary Table 3. We first calculated cumulative incidence curves,[6] using death as a competing event for comparing quartiles of cardiovascular age-delta. This is a descriptive analysis which does not include any covariates. We note that no clear split of the curves is visible, indicating that, in an unadjusted analysis, there is no clear association of cardiovascular age-delta with prospective MACE (see Supplementary Figure 8a).

Next, we assessed the association with a Cox model. We consider a fully covariate-adjusted model (age, sex, obesity, diabetes, smoking, alcohol, consumption, hypertension, coronary artery disease) and a minimally covariate-adjusted model (age, sex). MACE prior to the MRI visit are taken into account by a stratified analysis. We compare the lowest and highest quartile (Supplementary Figure 8b) and modelled cardiovascular age-delta as a continuous variable. Although the quartile comparison of the most extreme quartiles yields significant results (hazard ratio 1.09, $P = 0.017$), the association does not hold if we model cardiovascular age-delta as a continuous variable. Considering the cumulative incidence plot (Supplementary Figure 8a), this may be due to a inconsistent pattern across the quartiles. Quartile 2 shows the highest risk for MACE as well as in the fully covariate-adjusted model (Supplementary Figure 8c). Therefore, we note that additional analysis are required for strengthening the observed risk profiles of participants with low and high cardiovascular age-delta, and would likely be complemented by longer follow-up data.

Associations of cardiovascular age-delta with self-reported medication: We used data in 27,546 participants with complete data on commonly used drugs (Supplementary Table 5). We considered drugs for which effectiveness on clinical outcomes is well established: all major antihypertensives, i.e. beta blockers, angiotensin-converting-enzyme inhibitors (ACEi), angiotensin receptor blockers (ARBs), calcium channel blockers (CCBs) and diuretics, as well as statins, metformin and digoxin. .

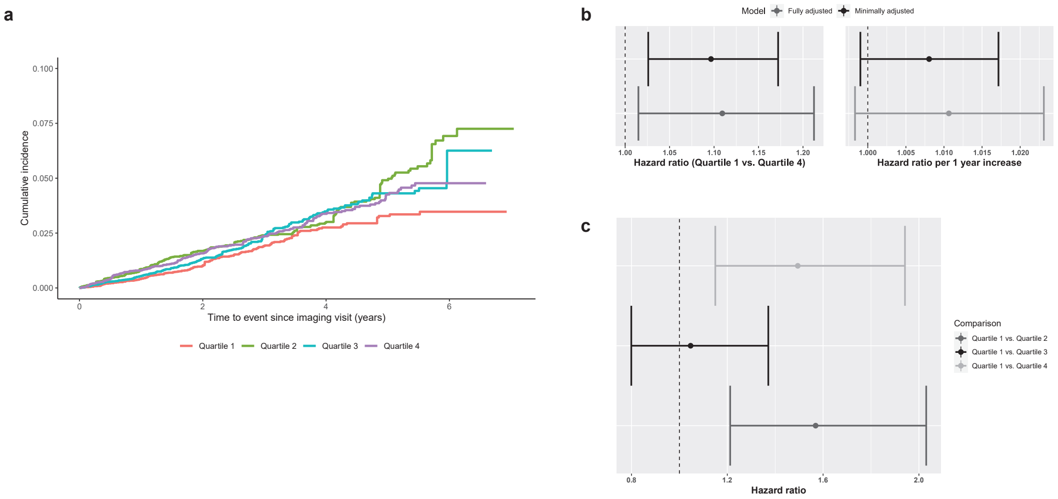

Supplementary Figure 8. Relationship of cardiovascular age-delta and outcomes. **a**, Cumulative incidence curves for time-to-first major adverse cardiovascular event, split into quartile groups based on cardiovascular age-delta. Figure is cut at 97.5 % percentile of reported survival times. **b**, Comparison of first and fourth quartile in a fully adjusted and minimally adjusted model and change in risk per 1 year change in cardiovascular age-delta (error bars indicate the hazard ratio point estimates ± 95% confidence intervals (CI) of the experiments). **c**, Comparison of quartiles of cardiovascular age-delta. The respective quartile groups are compared to the first quartile which consists of participants with lowest cardiovascular age-delta (error bars indicate the hazard ratio point estimates ± 95% confidence intervals (CI) of the experiments). For all experiments, n = 34,145.

Characteristic					*P value*
Quartile (age-delta range, years)	Quartile 1 (-32.4, -5.3)	Quartile 2 (-5.3, 0.2)	Quartile 3 (0.2, 5.3)	Quartile 4 (5.3, 31.9)	
N	6882	6882	6882	6882	
Diuretics	251 (3.6%)	316 (4.6%)	314 (4.6%)	346 (5.0%)	$9.6x10^{-4}$
CCB	543 (7.9%)	736 (11%)	742 (11%)	752 (11%)	$1.67x10^{-10}$
ARB	338 (4.9%)	383 (5.6%)	452 (6.6%)	468 (6.8%)	$2.69x10^{-6}$
ACEi	520 (7.6%)	626 (9.1%)	599 (8.7%)	665 (9.7%)	$1.33x10^{-4}$
BB	481 (7.0%)	498 (7.2%)	490 (7.1%)	448 (6.5%)	0.36
Digoxin	8 (0.1%)	8 (0.1%)	9 (0.1%)	11 (0.2%)	0.88
Metformin	188 (2.7%)	231 (3.4%)	243 (3.5%)	302 (4.4%)	$2.82x10^{-6}$
Statins	1,497 (22%)	1,686 (24%)	1,838 (27%)	1,670 (24%)	$5.38x10^{-10}$
Sex					$1.19x10^{-10}$
Female	3,679 (53%)	3,476 (51%)	3,391 (49%)	3,282 (48%)	
Male	3,203 (47%)	3,406 (49%)	3,491 (51%)	3,600 (52%)	
Chronological age	63 (56, 69)	65 (58, 71)	66 (60, 71)	64 (59, 69)	$2.38x10^{-84}$

Supplementary Table 5. Prevalence of self-reported medications across cardiovascular age-delta quartiles. This table shows the prevalences of self-reported drug use across cardiovascular age-delta quartiles. *N* denotes the number of participants in the corresponding quartile. *P*-values for drugs and sex were determined based on Pearsons Chi-squared test. The *P*-values for age and age-delta were calculated based on one-sided Kruskal-Wallis rank sum test. The table is derived on the complete-case set of participants used in the linear regression analyses of medication effects. Abbreviations: CCB, calcium channel blockers; BB, beta-blockers; ARB, angiotensin receptor blockers; ACEi, angiotensin converting enzyme inhibitors.

We used two linear regression models to investigate the association between drug use and age-delta. The rationale behind the additional adjustment in Model 2 is inclusion of covariates influenced by hypertensive medications with well established favourable effects on cardiovascular outcomes.

Model 1:

$$\text{Age delta} \sim \text{Drug prescribed (e.g. Metformin)} + \text{Age} + \text{Age}^2 + \text{Sex} + \text{Smoking pack years}$$
$$+ \text{Alcohol intake, grams per day} + \text{Body Mass Index} + \text{Obesity diagnosis} + \text{Coronary artery disease diagnosis}$$
$$+ \text{Hypertension diagnosis} + \text{Diabetes Mellitus diagnosis} + \text{Hypercholesterolaemia diagnosis} + \text{Heart Failure diagnosis}$$

Model 2:

$$\text{Age delta} \sim \text{Drug prescribed (e.g. Metformin)} + \text{Age} + \text{Age}^2 + \text{Sex} + \text{Smoking pack years}$$
$$+ \text{Alcohol intake, grams per day} + \text{Body Mass Index} + \text{Obesity diagnosis} + \text{Coronary artery disease diagnosis}$$
$$+ \text{Hypertension diagnosis} + \text{Diabetes Mellitus diagnosis} + \text{Hypercholesterolaemia diagnosis} + \text{Heart Failure diagnosis}$$
$$+ \text{Diastolic blood pressure} + \text{Systolic blood pressure} + \text{Heart Rate}$$

Model 1 had $R^2 = 0.11$, which reduced to $R^2 = 0.03$ in Model 2 after adjusting for heart rate and blood pressure.

Genotyping and whole exome analysis

Quality Control of genetic data: We used imputed genotypes and exome data as described by UK Biobank.[7–9] Participants of European ancestry only have been included and we followed quality control (QC) procedures recommended by UK Biobank, excluding participants as follows:

- Participants who withdrew consent for further analyses were removed (n=5).
- Outliers for heterozygosity or missing rate were removed (UK Biobank field 22027) (n=64).
- Discordance between genetically determined sex and self-reported sex or high missing rate (UK Biobank field 22001 and 31) (n=24).
- Participants with sex chromosome aneuploidy (UK Biobank field 22019) (n=18).
- Close relationships to others, determined by kinship coefficients by UK Biobank > 0.884 (n=2837). One of each pair was excluded at random.

We thus obtained 39,559 participants with CMR data, of which 37,177 participants were of European origin. Applying the above QC filter criteria left 29,506 participants.

We additionally performed a GWAS for ECG-predicted cardiovascular age-delta. Of 43,923 participants with complete ECG 12-lead trace data, 41,122 participants were of European ancestry. Following application of the above QC criteria, 31,475 participants remained.

Ancestry estimation: Hidden population substructure is a source of confounding hence we only included participants to comprise a well-mixed population. The majority of participants in the UK Biobank self-identify as white European. Using the R package `plinkQC` (v0.3.3), we used HapMap III reference data for ancestry estimation.[10] HapMapIII reference data was downloaded from ftp://ftp.ncbi.nlm.nih.gov/hapmap/genotypes/2009-01_phaseIII/plink_format/, joined to UK Biobank genotype data and principal component analysis performed. Participants that clustered with HapMap III participants of European ancestry of the PC1/PC2 plot were retained for analyses.

SNP heritability computation using Linkage Disequilibrium score regression: To estimate heritability index (h^2) we use the Linkage Disequilibrium (LD) score regression tool ("LDSC") obtained from https://github.com/bulik/ldsc. As input we used our GWAS summary statistics for the CMR and ECG GWASs and compute heritability as described in the wiki page https://github.com/bulik/ldsc/wiki/Heritability-and-Genetic-Correlation. We obtain $h^2 = 10.5\%$ for CMR summary statistics, and $h^2 = 10.4\%$ for ECG summary statistics.

Evidence for variant annotations: Genome wide significant loci were mapped to estimated causal genes using the following criteria:

- Using VEP annotation, assessment of evidence that the variant directly affects the function of a candidate gene (i.e. predicts damage or loss of function).

- Assessment of whether the variant is an eQTL for the candidate gene in a biologically relevant cell class or tissue. The presence of a colocalising e/pQTL was determined by searching the following resources: GTEx, CEDAR, Fairfax 2012, TWINSUK, eQTLGen, GENCORD, HIPSCI, GEUVADIS, Alasso 2018, Nedelec 2016, Blueprint, Quach 2016, Naranbhai 2015, Van de Bunt 2015, and Schwartzentruber 2018. Locus full summary statistics were downloaded for possible colocalising eQTLs.
- Evidence of additional functional data linking the variant to the gene, using https://genetics.opentargets.org/
- Assessment of the candidate gene's function and plausible contribution to cardiovascular ageing, with further review of Phenome Wide Association Study (PheWAS) associations.

Based on this criteria, the following variant-to-gene mappings were made for the 5 genome wide significant loci (see Supplementary Figure 9 for co-localisation locus plots):

- **rs2042995** eQTL data strongly suggest that the causal gene here is *TTN*. The lead variant, rs2042995, a missense variant found within the *TTN* coding sequence, is also associated with *TTN* expression in blood, atrial appendage, coronary artery, aorta and tibial artery. https://genetics.opentargets.org/variant/2_178693639_T_C

  Summary: Effect variant; increases cardiovascular age delta, probable causal gene: *TTN*.
- **rs7795735** This is an intergenic variant located 12.6 kilobases downstream of *ELN*, the gene that encodes elastin. This was a lead SNP associated with ascending aortic distensibility in a recent publication[11].

  Summary: Effect variant; increases cardiovascular age delta, probable causal gene: *ELN*.
- **rs1991860** This is an intronic variant for *PI15*, which is an eQTL for *PI15* in the tibial artery (GTEx). *PI15* is the closest gene to the locus. *PI15* encodes the peptidase inhibitor 15, which is associated with refractive errors and lung function (https://platform.opentargets.org/target/ENSG00000137558/associations/). There are no clear mechanistic links between the gene and cardiovascular ageing so the conclusion here is inconclusive.

  Summary: Effect variant; decreases cardiovascular age delta. Probable causal gene: *PI15*.
- **rs61886308** This is an intronic variant for *PLCE1*, and is also an eQTL for *PLCE1* in the coronary and tibial artery (GTEx) (Supplementary Figure 9d). *PLCE1* is the closest gene to the locus. Phospholipase C epsilon modulates beta-adrenergic receptor-dependent cardiac contraction and it has been found that this protein is over expressed during heart failure. https://genetics.opentargets.org/variant/10_94146109_G_A

  Summary: Effect variant; increases cardiovascular age delta. Probable causal gene: *PLCE1*.
- **rs2986036** This is an intronic variant for *NEURL1-AS1*, and is also an eQTL for *NEURL1-AS1* in the testes and fibroblasts (GTEx) (Supplementary Figure 9e). *NEURL1-AS1* is the closest gene to the locus. *NEURL1-AS1* was found to be associated with platelet aggregation response to adrenaline.[12] .

  Summary: Effect variant; decreases cardiovascular age delta. Possible causal gene: *NEURL1-AS1*.

Rare variants gene burden analysis: Table 1 shows that significant effect on the age-delta phenotype was found for two genes *TREM2* ($P = 5.0 \times 10^{-6}$, AF<0.1%) and *MICU3* ($P = 7.1 \times 10^{-6}$, on singletons). Leave-one-out-variant (LOVO) test was performed on *TREM2* (Supplementary Table 6). Out of the six LoF variants found in the gene, 6:41158691:C:T (rs2234258), a stop-gained variant, caused the association.

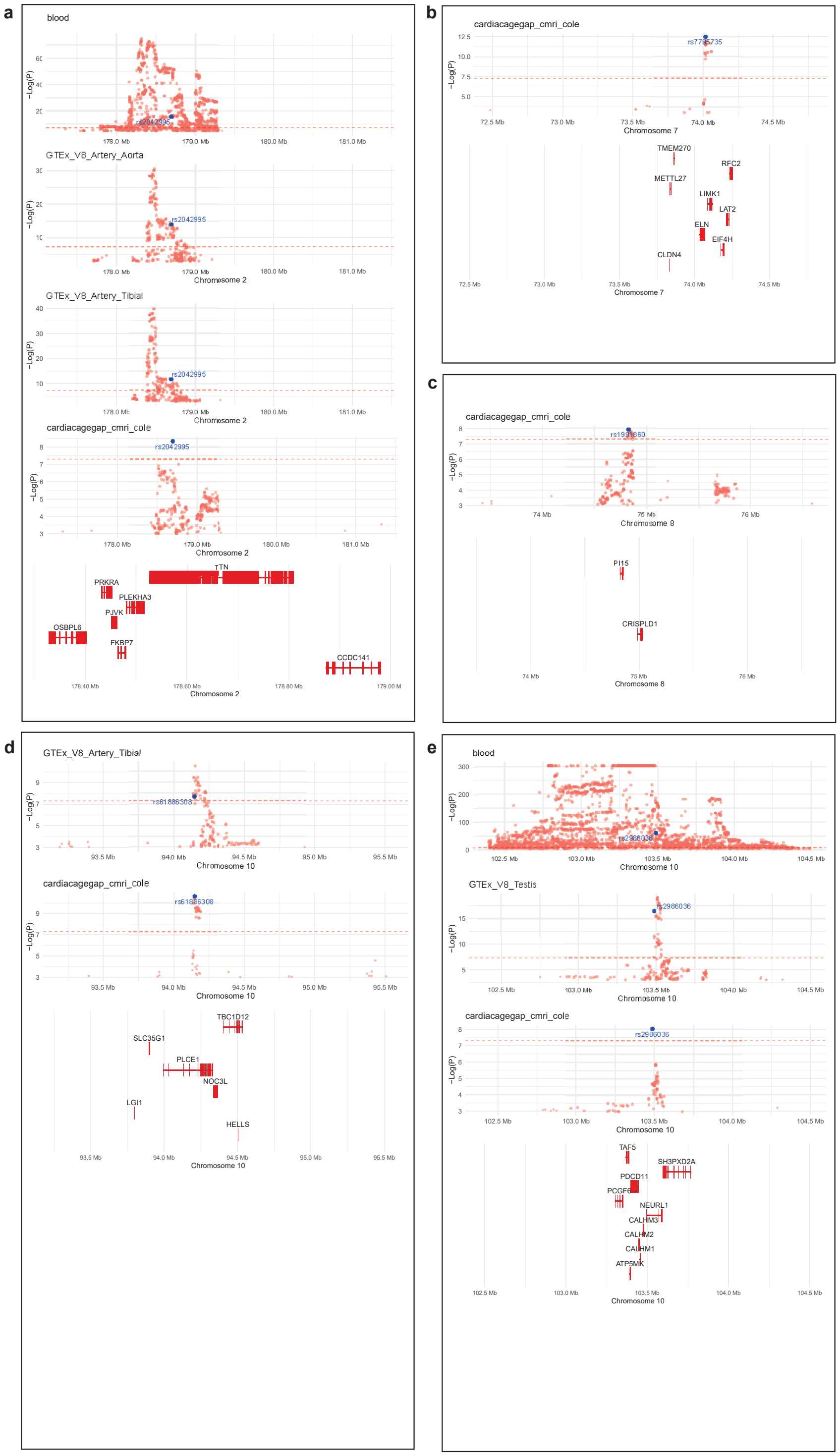

Supplementary Figure 9. Co-localisation plots of GWAS and eQTL signals at the 5 significant loci. Co-localisation plots of GWAS and eQTL signals at the 5 significant loci For each of the significant loci, eQTL signals of probable causal genes and some neighboring genes is shown.

Supplementary Table 6. *TREM2* leave-one-variant-out (LOVO) test. The LOVO scheme builds masks by leaving out one variant at a time from the full set of sites, and performs burden tests on each mask. Same as in Table 1, these are one-sided tests using whole genome regression modelling on additive effects of variants included in the mask. The significance level is set by multiple tests adjustment at: 0.05/10431 = 4.79x10^{-6} (negative LOG10P = 5.32).

Chromosome	Position	ID	A1FREQ	BETA	SE	LOG10P
6	41158691	*TREM2*(ENSG00000095970).M1.0.001_6:41158691:C:T	7.9×10^{-5}	0.34	3.36	0.04
6	41158994	*TREM2*(ENSG00000095970).M1.0.001_6:41158994:G:A	5.7×10^{-4}	5.61	1.27	5.00
6	41158639	*TREM2*(ENSG00000095970).M1.0.001_6:41158639:GT:G	5.7×10^{-4}	5.72	1.27	5.18
6	41159883	*TREM2*(ENSG00000095970).M1.0.001_6:41159883:C:T	5.7×10^{-4}	5.75	1.27	5.23
6	41158607	*TREM2*(ENSG00000095970).M1.0.001	5.9×10^{-4}	5.74	1.25	5.35
6	41158662	*TREM2*(ENSG00000095970).M1.0.001_6:41158662:G:A	5.7×10^{-4}	6.16	1.27	5.91
6	41161557	*TREM2*(ENSG00000095970).M1.0.001_6:41161557:G:A	5.7×10^{-4}	6.25	1.27	6.10

Polygenic risk score

The polygenic risk score (PRS) was constructed using 29,506 unrelated, European study participants, using SNPs with $P < 0.001$, clumped with LD threshold of $R^2 = 0.1$, window of 250kb. We then constructed a linear (null) model predicting the age-delta using the sex, age (at time of MRI), age^2, MRI assessment centre and genotyping array as covariates. R^2 of the null model was 0.01. This is compared to a model that adds PRS as an additional covariate. The fraction of variance explained by PRS (Supplementary Table 7) is reported as ΔR^2, and equates to the difference of R^2 between these two models.

Supplementary Table 7. Results of polygenic risk score (PRS) construction for cardiovascular age-delta using various single nucleotide polymorphism inclusion significance thresholds, with corresponding fraction of variance explained by PRS ΔR^2.

P-value SNP inclusion threshold	Number of SNPs used	ΔR^2
1×10^{-6}	13	0.01
1×10^{-5}	45	0.03
1×10^{-4}	284	0.11
1×10^{-3}	1845	0.35
1×10^{-2}	13592	0.66

PRS age-delta PheWAS results

In 415,622 genotyped European ancestry UK Biobank participants, the age-delta PRS was positively associated with a diagnosis of hypertension (OR 1.005, $P = 1.1 \times 10^{-11}$) and systolic (OR 1.01, $P = 9.3 \times 10^{-13}$) and diastolic (OR 1.01, $P = 5.3 \times 10^{-11}$) blood pressure. Coeliac disease was also a significant negative association (Supplementary Figure 10).

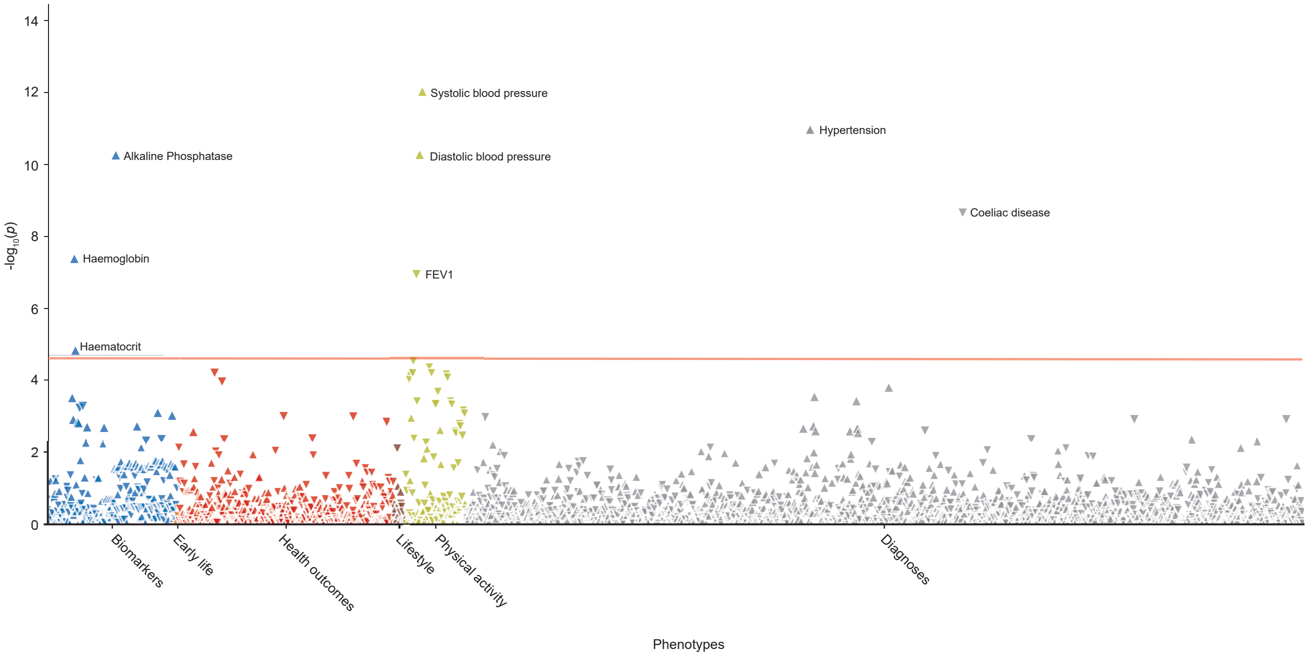

Supplementary Figure 10. PheWAS using PRS of cardiovascular age-delta. Phenome wide analysis of age-delta PRS adjusted for age, age^2, sex and the first ten genetic principal components by two-sided logistic regression ($n = 415{,}622$ genotyped White British UK Biobank participants). The red line represents the significance threshold after accounting for multiple testing using Bonferroni correction (2407 traits, $P < 2.1 \times 10^{-5}$). Upright triangles indicate positive associations, and the inverted triangles indicate negative associations. FEV1, forced expiratory volume in first second.

Age prediction using resting electrocardiograms

Model specification: We used a convolutional residual neural network architecture,[13] to predict the age of UK Biobank participants, given as input the corresponding resting electrocardiogram (ECG) traces. The model code and weights were obtained from https://github.com/antonior92/ecg-age-prediction.

Data preparation: UK Biobank resting ECG traces were parsed from the XML files provided on the Research Analysis Platform. In these data, one sample corresponds to a measured trace for 1 second at 500 Hz. We downsampled to 400 Hz by uniformly selecting 400 data points for each sample. Afterwards, we selected 4096 data points starting from sample 2. Input data were scaled to be on 1e-4 V scale by dividing the input traces by 10. This preprocessing meets the required input format for the algorithm.

Training the model: To adapt the prediction model to the UK Biobank traces, we performed a series of fine-tuning runs. We used our defined healthy participants (see Methods) for training. Performance is evaluated on a held-out validation set of 1000 participants in each training run (roughly 20 percent of the samples). Supplementary Table 8 shows the model-parameters that were used. We present the training results across six different training settings, starting with the pretrained model weights without refinement, full retraining with random and pretrained weights, and fine-tuning of 3 million, 1.7 million and 5120 parameters from the last layers in the network architecture. The models' prediction performances, assessed as Pearson correlation coefficient ($|r|$) between predicted age and chronological age at the imaging visit are shown in Supplementary Table 9. Training and validation loss of our selected model **model_pretrained_v3** are shown in Supplementary Figure 11.

Supplementary Table 8. Model parameters for ECG model fine-tuning. For the training procedure we used these model parameters and left all other parameters as set by default in the ECG model program.

	epochs	random_seed	sample_freq	seq_length	batch_size	lr	patience	min_lr	lr_factor	net_filter_size	net_seq_lengh	dropout_rate	kernel_size
1	50	112233	400	4096	32	0.001	7	1e-07	0.1	64,128,196,256,320	4096,1024,256,64,16	0.8	17

Supplementary Table 9. Fine-tuning results for ECG training. The model architecture was retrained in different ways on healthy UK Biobank participants. Above we defined 5066 participant as a 'healthy' population, from which ECG traces from 4501 participants were available. Performance was assessed by correlating the chronological age of the participants at the imaging visit to the predicted age from the model. We perform Cole's method of age-bias correction as described for image-based age prediction described above.[14] Columns presented in this table are: *r_NoCorrection* - Pearson correlation of raw model age predictions to chronological age of participants at imaging visit. *r_ColeCorrected* - Pearson correlation of age-bias corrected age predictions to chronological age. *AgeRange_NoCorrection* - range of predicted ages, no age bias correction. *AgeRange_ColeCorrected* - range of predicted ages with age-bias correction. *AgeRange* - Range of chronological ages at imaging visit. *mean_pred_age* - Mean of predicated ages, *mean_delta* - Mean of predicted 'age-delta', i.e. Predicted age minus chronological Age. We selected a model **model_pretrained_v3** to perform predictions in the rest of UK Biobank.

ModelId	Description	r_NoCorrection	r_ColeCorrected	AgeRange_NoCorrection	AgeRange_ColeCorrected	AgeRange	mean_pred_age	mean_age	mean_delta
model_notrain	Lima et.al. original model	0.31	0.49	-23.2-88.1	2.8-121.4	46-81	34.60	61.80	-27.20
model_fulltrain_v3	Full retrain on 5K, random weights	0.07	0.84	50-73.9	37.4-86.7	46-81	63.90	61.80	2.10
model_fulltrain_v2	Full retrain on 5K, Lima et.al. weights init	0.54	0.80	40.6-86.6	34-93.3	46-81	62.50	61.80	0.70
model_pretrained_v5	Fine tune 3 Mio parameters from Lima	0.50	0.83	40.6-81.3	29.4-90.6	46-81	63.40	61.80	1.60
model_pretrained_v3	Fine tune 1.7 Mio parameters from Lima	0.50	0.83	40.6-81.2	29.4-90.6	46-81	63.40	61.80	1.60
model_pretrained_v4	Fine tune 5120 parameters from Lima	0.50	0.62	31.4-89.2	26.4-92.7	46-81	62.40	61.80	0.60

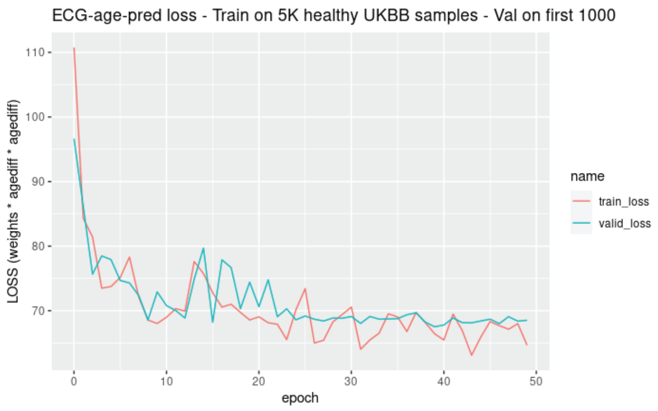

Supplementary Figure 11. Training and validation loss of the fine-tuning of our selected ECG model **model_pretrained_v3**.

Prediction performance of ECG age-gap predictions in healthy participants: As described above, we train our model using 4501 participants, from which we defined a held-out validation set of 1000 random participants serving as performance indicator. We assess the model's performance by the $|r|$ of the predicted vs. chronological age in these 1000 validation participants. Supplementary Figure 12 shows the scatter plots with the predictions. We observe similar prediction performance as the image-based CatBoost model ($|r| = 0.50$, Supplementary Table 9) before age bias correction, and a strong dependency of the age deltas on age, as reported previously. After correction for age-bias,[14] Pearson correlation of chronological age vs. predicted age increases to $|r| = 0.85$ in the healthy validation participants, and age dependency disappears ($r = -0.01$). This is very similar to the results of the image-based CatBoost age delta predictions.

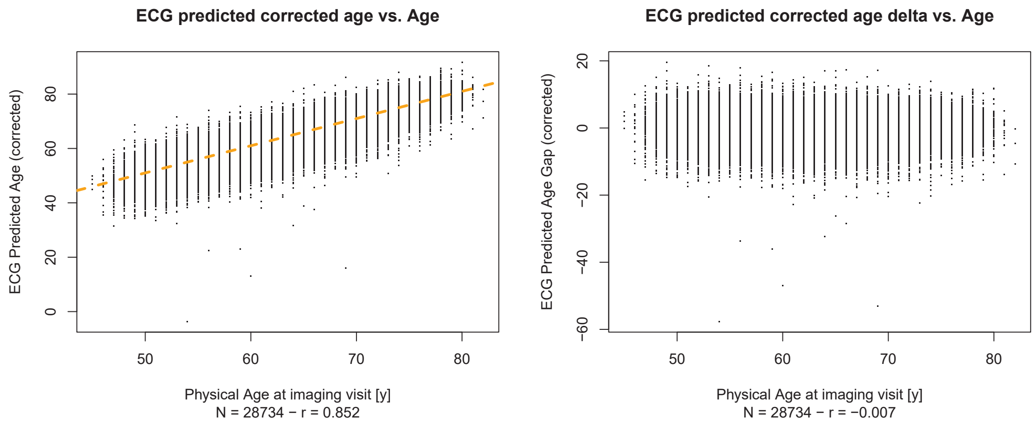

Supplementary Figure 12. Training performance assessed as Pearson correlation of predicted vs. chronological age in 1000 healthy held-out participants, using our selected ECG model **model_pretrained_v3**.

Prediction of age from ECGs in UK Biobank: Given our refined model **model_pretrained_v3** we predict on the remaining 34,108 UK Biobank participants, which, by our definition, do not belong to the "healthy" group. For comparison to image-based predictions, we show the intersection of the 34,108 ECG-based age predictions versus the 34,147 image-based age predictions, leaving in total 28,734 participants. We observe a correlation in the predicted ages (Pearson $|r| = 0.64$), while there is no correlation in age-deltas ($|r| = 0.01$). Supplementary Figure 13 shows the results as scatterplots.

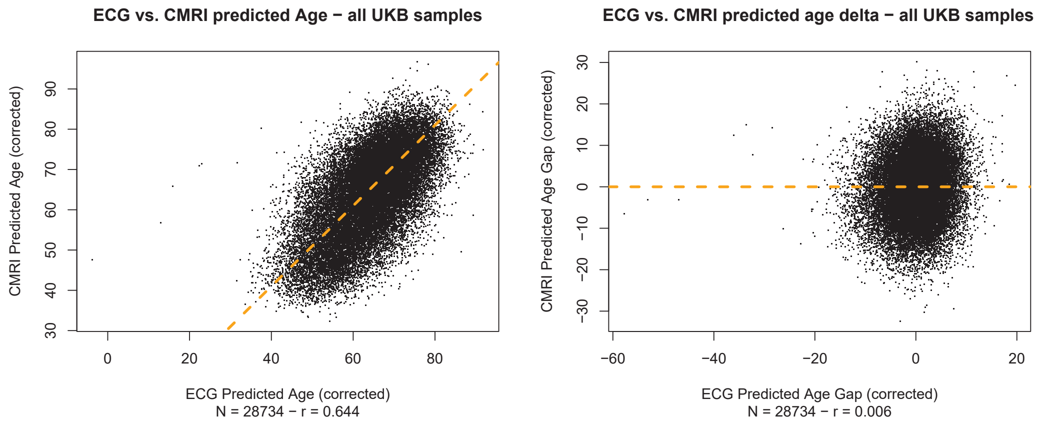

Supplementary Figure 13. Comparing prediction of 28734 predicted UK Biobank participants using cardiac magnetic resonance imaging and electrocardiogram (ECG)-based prediction models.

GWAS for ECG predicted age-delta: Analogous to the GWAS for the CMR predicted age-delta we ran a genome wide association study for the ECG age-delta prediction using PLINK workflow on DNANexus (https://platform.dnanexus.com/app/plink_gwas, v1.0.6). We used imputed genotypes in the Bulk/Imputation/UKBiobankimputationfromgenotype folder with the following options:

```
--covar-name Sex,Age,Array,PC1-PC10
--covar-variance-standardize
--geno 0.03
--glm hide-covar
--maf 0.01
--mind 0.05
```

Outputs were collected for each chromosome and a Manhattan plot was generated using a custom ggplot with R. The Manhattan plot is shown in Figure 8 and the associated loci in Table 1. The following variant-to-gene mappings were made for the 8 genome wide significant loci.

Variant annotations:

- **rs4671961** (*EML6*) Summary: Closest gene is *SPTBN1*, but variant is an eQTL for further apart gene *EML6* in tibial, coronary and artery aorta. (https://genetics.opentargets.org/variant/2_54571459_G_A)
- **rs11902709** (*TTN*) Intron variant for *TTN* (closest gene). Identified in both ECG and CMRI based age-delta prediction GWASes. pQTL for proteins FKBP7. PheWAS links to pulse rate and AV-block. (https://genetics.opentargets.org/variant/2_178743480_C_T)
- **rs7373065** (*SCN5A*) *SCN5A* is the closest gene. Other close locus genes are e.g. *SCN10A* and *EXOG*. PheWAS links variant to atrial fibrillation, pulse rate, cardiac arrhythmias. (https://genetics.opentargets.org/variant/3_38668824_T_C)
- **rs35430511** (*CAMK2D*) Closest gene is *CAMK2D*, promoter link in PCHi-C.[15] PheWAS links to atrial fibrillation. (https://genetics.opentargets.org/variant/4_113465982_T_C)
- **rs147790633** (*MYOZ1*) Closest gene is *AGAP5*. eQTL in heart atrial appendage for *MYOZ1*. *MYOZ1* has been implicated in a heart failure GWAS and encodes Z-disc cardiac binding proteins. PheWAS links to atrial fibrillation, cardiac arrhythmias, forced expiratory volume. (https://genetics.opentargets.org/variant/10_73687824_T_C)
- **rs60820984** (*CAMK2G*) Closest gene is *CAMK2G*, which encodes a protein that regulates intracellular calcium signalling. Close to rs147790633, and also shows eQTL for *MYOZ1* in heart atrial appendage as this SNP. Linked to promoter in PCHi-C,[15] and DNAse hypersensitive sites.[16] Further linked as enhancer for *NDST2* (*FANTOM5*). *CAMK2G* is a known target for the prognostic drug Empagliflozin[17]. PheWAS links to atrial fibrillation, cardiac arrhythmias, peek expiratory flow and lung function. (https://genetics.opentargets.org/variant/10_73879820_C_T)
- **rs7132327** (*TBX3*) Intergenic variant, closest gene is *TBX3* at around 260kb distance. TBX3 is a critical transcription factor for the development of the conduction system, as well as regulation of its structure and function[18]. PheWAS links to myocardial fractal dimension (https://genetics.opentargets.org/variant/12_114943266_T_C)
- **rs35866366** (*SIPA1L1*) eQTL in immune cells and blood and placenta, closest gene *SIPA1L1*. Link to promoter in PCHi-C.[15] PheWAS links to benign neoplasm of other parts of digestive system and myocardial fractal dimension (https://genetics.opentargets.org/variant/14_71382468_A_G)

Supplementary References

1. van Buuren, S. Multivariate imputation by chained equations. R package version 3.11.0. https://www.rdocumentation.org/packages/mice/versions/3.14.0/topics/mice/ (2020).
2. de Lange, A.-M. G. *et al.* Mind the gap: Performance metric evaluation in brain-age prediction. *Hum Brain Mapp* **43**, 3113–3129 (2022).
3. Evangelou, E. *et al.* Alcohol consumption in the general population is associated with structural changes in multiple organ systems. *eLife* **10**, e65325 (2021).
4. Ho, D. E., Imai, K., King, G. & Stuart, E. A. MatchIt: Nonparametric preprocessing for parametric causal inference. *J Stat Softw* **42**, 1–28 (2011).
5. Benjamini, Y. & Hochberg, Y. Controlling the false discovery rate: A practical and powerful approach to multiple testing. *J R Stat Soc Series B Stat Methodol* **57**, 289–300 (1995).
6. Gray, R. J. A class of K-sample tests for comparing the cumulative incidence of a competing risk. *Ann Stat* 1141–1154 (1988).
7. Bycroft, C. *et al.* The UK Biobank resource with deep phenotyping and genomic data. *Nature* **562**, 203–209 (2018).
8. Van Hout, C. V. *et al.* Exome sequencing and characterization of 49,960 individuals in the UK Biobank. *Nature* **586**, 749–756 (2020).
9. Szustakowski, J. D. *et al.* Advancing human genetics research and drug discovery through exome sequencing of the UK Biobank. *Nat Genet* **53**, 942–948 (2021).
10. Altshuler, D. M. *et al.* Integrating common and rare genetic variation in diverse human populations. *Nature* **467**, 52–8 (2010).
11. Francis, C. M. *et al.* Genome-wide associations of aortic distensibility suggest causality for aortic aneurysms and brain white matter hyperintensities. *Nat Commun* **13**, 1–18 (2022).
12. Johnson, A. D. *et al.* Genome-wide meta-analyses identifies seven loci associated with platelet aggregation in response to agonists. *Nat Genet* **42**, 608–613 (2010).
13. Lima, E. M. *et al.* Deep neural network-estimated electrocardiographic age as a mortality predictor. *Nat Commun* **12**, 5117 (2021).
14. de Lange, A.-M. G. & Cole, J. H. Commentary: Correction procedures in brain-age prediction. *Neuroimage Clin* **26**, 102229 (2020).
15. Javierre, B. M. *et al.* Lineage-Specific Genome Architecture Links Enhancers and Non-coding Disease Variants to Target Gene Promoters. *Cell* **167**, 1369–1384.e19 (2016).
16. Thurman, R. E. *et al.* The accessible chromatin landscape of the human genome. *Nature* **489**, 75–82 (2012).
17. Shao, G. Pharmacological analysis of Empagliflozin: Acting through the CaMKII pathway in type 2 diabetes and acute cardiovascular events. *PLoS One* **17**, 1–12 (2022).
18. Mohan, R. A. *et al.* T-box transcription factor 3 governs a transcriptional program for the function of the mouse atrioventricular conduction system. *Proc Natl Acad Sci* **117**, 18617–18626 (2020).
