# Supplementary material for: Environmental and genetic predictors of human cardiovascular ageing
Source: Nat Commun. 2023 Aug 21;14:4941. doi: 10.1038/s41467-023-40566-6 (PMC10442405; doi:10.1038/s41467-023-40566-6)
Supplement: Supplementary file 3 — Reporting Summary [file 41467_2023_40566_MOESM3_ESM.pdf]

nature portfolio

Corresponding author(s): Declan P. O'Regan

Last updated by author(s): Jul 14, 2023

Reporting Summary

Nature Portfolio wishes to improve the reproducibility of the work that we publish. This form provides structure for consistency and transparency in reporting. For further information on Nature Portfolio policies, see our Editorial Policies and the Editorial Policy Checklist.

Statistics

For all statistical analyses, confirm that the following items are present in the figure legend, table legend, main text, or Methods section.

n/a	Confirmed	
☐	☒	The exact sample size (*n*) for each experimental group/condition, given as a discrete number and unit of measurement
☐	☒	A statement on whether measurements were taken from distinct samples or whether the same sample was measured repeatedly
☐	☒	The statistical test(s) used AND whether they are one- or two-sided<br>*Only common tests should be described solely by name; describe more complex techniques in the Methods section.*
☐	☒	A description of all covariates tested
☐	☒	A description of any assumptions or corrections, such as tests of normality and adjustment for multiple comparisons
☐	☒	A full description of the statistical parameters including central tendency (e.g. means) or other basic estimates (e.g. regression coefficient) AND variation (e.g. standard deviation) or associated estimates of uncertainty (e.g. confidence intervals)
☐	☒	For null hypothesis testing, the test statistic (e.g. *F*, *t*, *r*) with confidence intervals, effect sizes, degrees of freedom and *P* value noted<br>*Give P values as exact values whenever suitable.*
☒	☐	For Bayesian analysis, information on the choice of priors and Markov chain Monte Carlo settings
☒	☐	For hierarchical and complex designs, identification of the appropriate level for tests and full reporting of outcomes
☐	☒	Estimates of effect sizes (e.g. Cohen's *d*, Pearson's *r*), indicating how they were calculated

Our web collection on statistics for biologists contains articles on many of the points above.

Software and code

Policy information about availability of computer code

Data collection	R v.>3.6.0 and Python v.3.9 (python package Optuna was used in hyperparameter search when refining CatBoost model).<br>GWAS analyses for cardiovascular age-delta were performed with PLINK (v.1.9 and later).<br>UK Biobank Research Analysis Platform (RAP).<br>MICE package for R, v3.16.0
Data analysis	Analysis code documented at https://github.com/ImperialCollegeLondon/cardiovascular_ageing

For manuscripts utilizing custom algorithms or software that are central to the research but not yet described in published literature, software must be made available to editors and reviewers. We strongly encourage code deposition in a community repository (e.g. GitHub). See the Nature Portfolio guidelines for submitting code & software for further information.

Data

Policy information about availability of data

All manuscripts must include a data availability statement. This statement should provide the following information, where applicable:

- Accession codes, unique identifiers, or web links for publicly available datasets
- A description of any restrictions on data availability
- For clinical datasets or third party data, please ensure that the statement adheres to our policy

All raw and derived data in this study are available from UK Biobank (http://www.ukbiobank.ac.uk/), conducted under

application number 40616. GWAS summary level data are publicly available through the GWAS catalogue (https://www.ebi.ac.uk/gwas/), deposited using accession numbers GCST90239748 and GCST90239749. For colocalization analyses, we used the unfiltered eQTL results from eQTL Catalogue (https://www.ebi.ac.uk/eqtl/) and the Genotype-Tissue Expression (GTEx) Portal v.8 (https://gtexportal.org/home/)

Human research participants

Policy information about studies involving human research participants and Sex and Gender in Research.

Reporting on sex and gender	The term "sex" is used throughout the manuscript. Details of methods used to determine this attribute are detailed in "Supplementary Methods".
Population characteristics	Please see Extended Data Figure 1. "Baseline participant characteristics".
Recruitment	All individuals were recruited as part of UK Biobank (http://www.ukbiobank.ac.uk/).
Ethics oversight	Please see https://www.ukbiobank.ac.uk/learn-more-about-uk-biobank/about-us/ethics

Note that full information on the approval of the study protocol must also be provided in the manuscript.

Field-specific reporting

Please select the one below that is the best fit for your research. If you are not sure, read the appropriate sections before making your selection.

[x] Life sciences [ ] Behavioural & social sciences [ ] Ecological, evolutionary & environmental sciences

For a reference copy of the document with all sections, see nature.com/documents/nr-reporting-summary-flat.pdf

Life sciences study design

All studies must disclose on these points even when the disclosure is negative.

Sample size	The full dataset used in this study contained 39,559 individuals. 39,559 was the maximum number of available samples in UK Biobank to ensure sufficient power for our study.
Data exclusions	Details of the excluded samples can be found in Supplementary Material and pertained to the genetic analyses.<br>For the genetic studies, participants of European ancestry only have been included as per community standard, and we followed quality control (QC) procedures recommended by UK Biobank, excluding participants as follows:<br>• Participants who withdrew consent for further analyses were removed (n=5).<br>• Outliers for heterozygosity or missing rate were removed (UK Biobank field 22027) (n=64).<br>• Discordance between genetically determined sex and self-reported sex or high missing rate (UK Biobank field 22001 and 31) (n=24).<br>• Participants with sex chromosome aneuploidy (UK Biobank field 22019) (n=18).<br>• Close relationships to others, determined by kinship coefficients by UK Biobank > 0.884 (n=2837). One of each pair was excluded at random.<br>We thus obtained 39,559 participants with CMR data, of which 37,177 participants were of European origin. Applying the above QC filter criteria left 29,506 participants.<br>We additionally performed a GWAS for ECG-predicted cardiovascular age-delta. Of 43,923 participants with complete ECG 12-lead trace data, 41,122 participants were of European ancestry. Following application of the above QC criteria, 31,475 participants remained.
Replication	Analyses were based on single measurements for each individual subject and therefore, technical replicates are not present in the data. Reproducibility in our results was confirmed through the use of independent datasets (discovery and validation) for the genetic analyses.
Randomization	Disease groups were first defined by strict criteria, and the full sample used to maximise power. Control groups for these disease groups were randomly selected from a large pool and subsequently 1:1 propensity-matched to disease groups by age and sex to control for confounding factors.
Blinding	Blinding was not relevant to this study as this was not a case control study, and group allocation was not performed. The study involved predicting quantitative traits in a sample population. Assignment of subjects to the independent discovery and validation sets for GWAS analysis was determined based on date of release from UK Biobank.

Reporting for specific materials, systems and methods

We require information from authors about some types of materials, experimental systems and methods used in many studies. Here, indicate whether each material, system or method listed is relevant to your study. If you are not sure if a list item applies to your research, read the appropriate section before selecting a response.

Materials & experimental systems

n/a	Involved in the study
☒	☐ Antibodies
☒	☐ Eukaryotic cell lines
☒	☐ Palaeontology and archaeology
☒	☐ Animals and other organisms
☒	☐ Clinical data
☒	☐ Dual use research of concern

Methods

n/a	Involved in the study
☒	☐ ChIP-seq
☒	☐ Flow cytometry
☒	☐ MRI-based neuroimaging
